# Different pathways for preterm birth between singleton and twin pregnancies: a population‐based registry study of 481 176 nulliparous women

**DOI:** 10.1111/1471-0528.17344

**Published:** 2022-11-21

**Authors:** Tiril Tingleff, Sari Räisänen, Åse Vikanes, Leiv Sandvik, Meryam Sugulle, Gulim Murzakanova, Katariina Laine

**Affiliations:** ^1^ Faculty of Medicine, Institute of Clinical Medicine University of Oslo Oslo Norway; ^2^ Department of Obstetrics Oslo University Hospital Oslo Norway; ^3^ Tampere University of Applied Sciences Tampere Finland; ^4^ Norwegian Research Centre for Women's Health Oslo University Hospital Oslo Norway; ^5^ Gynklinikk Nydalen AS Oslo Norway

**Keywords:** epidemiology, extremely preterm birth, late preterm birth, preterm birth, twin pregnancy, very preterm birth

## Abstract

**Objective:**

To explore the contribution of pregnancy‐related complications on the prevalence of extremely, very and late preterm births in singleton and twin pregnancies. To study the risk of spontaneous preterm birth in twin pregnancies compared with singleton pregnancies.

**Design:**

Population‐based registry study.

**Setting:**

Medical birth registry of Norway and Statistics Norway.

**Population:**

Nulliparous women with singleton (*n* = 472 449) or twin (*n* = 8727) births during 1999–2018.

**Methods:**

Prevalence rates of pregnancy‐related complications for extremely, very and late preterm birth in twin and singleton pregnancies were calculated with 95% confidence intervals. Multivariable logistic regression was applied to assess odds ratios for preterm birth, adjusted for obstetric and socio‐economic factors.

**Main outcome measures:**

Extremely preterm (<28^+0^ weeks of gestation), very preterm (28^+0^–33^+6^ weeks of gestation) and late preterm (34^+0^–36^+6^ weeks of geatation) birth.

**Results:**

Preterm birth was significantly more prevalent in twin pregnancies than in singleton pregnancies in all categories: all preterm (54.7% vs 6.1%), extremely preterm (3.6% vs 0.4%), very preterm (18.2% vs 1.4%) and late preterm (33.0% vs 4.3%) births. Stillbirth, congenital malformation and pre‐eclampsia were more prevalent in twin pregnancies than in singleton pregnancies, but the prevalence of complications differed in the three categories of preterm birth. Pre‐eclampsia was more prevalent in singleton than in twin pregnancies ending in extremely and very preterm birth. The adjusted odds of spontaneous preterm live birth were between 19‐ and 54‐fold greater in twin pregnancies than in singleton pregnancies.

**Conclusions:**

Singleton and twin pregnancies seem to have different pathways leading to extremely, very and late preterm birth.

## Abstract 2

This article includes Author Insights, a video abstract available at: https://vimeo.com/manage/videos/775727344

## Graphical abstract

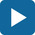

**Abbreviations**
aOR: adjusted odds ratio
ASA: acetylsalicylic acid
EEA: European Economic Association
HELLP: haemolysis, elevated liver enzymes and low platelet count
MBRN: The Medical Birth Registry of Norway
PROM: prelabour rupture of membranes
SSB: Statistics Norway

## 1 INTRODUCTION

Prematurity is a major contributor to neonatal morbidity and mortality. The incidence of preterm birth is higher in twin pregnancies than in singleton pregnancies: approximately 50% of twins are born before 37 weeks of gestation, accounting for 18%–25% of all preterm births.^1^ Physiological changes in pregnancy that strain maternal organ systems are more pronounced in twin pregnancies.^2^ Numerous maternal and fetal pregnancy‐related complications that may lead to preterm delivery, such as hypertensive disorders of pregnancy, congenital malformations and fetal demise, are more common in twin pregnancies than in singleton pregnancies.^3^, ^4^ Preterm birth is a complex syndrome, and the underlying mechanisms may differ between singleton and twin gestations.^5^, ^6^, ^7^, ^8^, ^9^ The extent of obstetric complications leading to preterm birth in twin pregnancies compared with singleton pregnancies is not well understood and has not been extensively studied.

We conducted this study to compare the distribution of pregnancy‐related complications representing different pathways leading to extremely, very and late preterm birth between singleton pregnancies and twin pregnancies.

We also assessed the risk of spontaneous preterm birth in twin pregnancies compared with singleton pregnancies with adjustment for known risk factors.

## 2 METHODS

### 2.1 Study population and design

For this population‐based registry study, data were obtained from two national registers, the Medical Birth Register of Norway (MBRN) and Statistics Norway (SSB), comprising all births occurring in Norway between 1999 and 2018. Notification to the MBRN for all births occurring in Norway has been mandatory since 1967. Information is obtained from antenatal health cards filled in at check‐ups and hospital birth medical records. All antenatal care in Norway is standardised and free of charge for residents. We collected data on maternal pre‐pregnancy health, pregnancy and birth from the MBRN. The SSB compiles official statistics about Norwegian residents, such as country of birth and education level, and we collected such data from this registry.

All nulliparous women with singleton or twin pregnancies who gave birth at gestational ages of ≥22 and ≤44 weeks during the study period were included, resulting in the inclusion of 481 176 women. Of these pregnancies, 472 449 were singleton pregnancies and 8727 were twin pregnancies. Pregnancy dating was based on routine ultrasound examinations performed in gestational weeks 17–20. When ultrasound dating was not available (2%), gestational age was estimated based on the first day of the woman's last menstruation.

### 2.2 Variables

The target outcome variable, gestational age at birth, was categorised into three groups: extremely preterm (before week 28), very preterm (weeks 28^+0^–33^+6^), and late preterm (weeks 34^+0^–36^+6^). The distinction between very preterm and late preterm was set at the timepoint after which neonatal outcomes are notably better. Norwegian clinical guidelines recommend treatment with tocolytics and corticosteroids for births occurring before week 34^+0^. We compared outcomes in twin and singleton pregnancies.

Factors known to be associated with preterm birth were examined. Stillbirth described fetal death before or during labour. Twin pregnancies were categorised as resulting in stillbirth if one or both twins were stillborn. Congenital malformations included all malformations registered in the MBRN (anencephaly, spina bifida, encephalocele, omphalocele, gastroschisis, congenital hydrocephalus, neural tube defects, diaphragmatic hernia, limb reduction defects, renal agenesis, anorectal atresia or stenosis, oesophageal atresia or stenosis, hypoplastic left heart and transposition of the cardiac great vessels). Twin pregnancies were categorised as involving congenital malformation if one or both twins were diagnosed with a malformation. Pre‐eclampsia (blood pressure > 140/90 mmHg and proteinuria); haemolysis, elevated liver enzymes and low platelet count (HELLP) syndrome; and eclampsia were merged into a single variable: pre‐eclampsia. Prelabour rupture of membranes (PROM) was defined as the rupture of membranes at least 12 h before the start of labour contractions. The onset of birth was defined as spontaneous (onset of spontaneous labour contractions, before intervention in women with PROM), induced (induction of contractions, after intervention in women with PROM) and caesarean section (caesarean delivery before the start of contractions), as listed in the MBRN.

The maternal country of birth was categorised using ten regions, based on the definitions used by the World Bank.^10^ Women born in Norway served as the reference group. European countries were divided into European Economic Association (EEA, including Switzerland) and non‐EEA countries. As few participants were from Transcaucasia and Central Asia, they were included in the non‐EEA group. Information on education from the SSB served as a proxy for socio‐economic status. The eight education levels listed in the 2011 International Standard Classification of Education were merged into four groups according to the years of highest completed education. Women who had completed secondary education served as the reference group. Marital status was categorised as married/cohabitating with partner and other. Maternal age at the time of birth was divided into 5‐year intervals, with separate categories for women aged <20 years and >40 years. Women aged 25–29 years served as the reference group. Information on smoking during the first trimester, registered in the MBRN, was recorded as never, sometimes, daily and missing. Diabetes was categorised as type 1, type 2 and gestational. In vitro fertilization was a dichotomous variable.

### 2.3 Missing data

Information on smoking was missing for 16.8% of the participants, and information on education was missing for 2.2% of the study population; these cases were categorised as missing in the analyses. Missing data rates for all other variables were <1%.

### 2.4 Ethical approval and patient involvement

This study had no patient involvement, as it was based on data from mandatory national registers. This study was part of The PURPLE Study, approved by the Regional Committee for Medical Research Ethics in South East Norway in 2015 (2015/681). Patient consent was not required. The Norwegian SIDS (sudden infant death syndrome) and Stillbirth Society provided financial support for the retrieval of data files from the registries (grant no. 554.04/14).

### 2.5 Statistical analysis

Descriptive statistics were used to calculate the prevalence (*n*, %) of maternal and obstetric characteristics. The analyses were conducted in a stepwise manner. First, the prevalence of extremely, very and late preterm and term births for women with twin and singleton pregnancies was calculated with 95% confidence intervals (95% CIs). Differences were considered significant when 95% CIs did not overlap. Second, the prevalence of the first factor associated strongly with extremely, very and late preterm births was calculated with 95% CIs separately for singleton and twin pregnancies. Third, cases with the first factor were excluded and the same procedure was conducted for the next strongly associated factor. Factors considered to be associated strongly with preterm birth were stillbirth, congenital malformations, pre‐eclampsia, PROM and non‐spontaneous onset of birth; spontaneous live births with no congenital malformation, pre‐eclampsia or PROM were included in the final analysis.

Multivariable regression analyses of the three preterm birth types were used to calculate crude odds ratios (ORs) and adjusted odds ratios (aORs) with 95% CIs separately. Only continuing pregnancies were included in the analysis, i.e. when analysing very preterm birth as an outcome, extremely preterm births were excluded, and when analysing late preterm birth as an outcome, extremely and very preterm births were excluded. Two models were established, with singleton pregnancies serving as the reference. Model 1 included the entire study population and model 2 included women with live spontaneous births with no congenital malformation, pre‐eclampsia or PROM. The assumptions underlying the multivariate logistic regression were found to be met adequately (all factors had variant inflation factors of <5).

IBM® SPSS® Statistics 27 (IBM, Armonk, NY, USA) was used to perform the statistical analyses.

## 3 RESULTS

**FIGURE 1:**
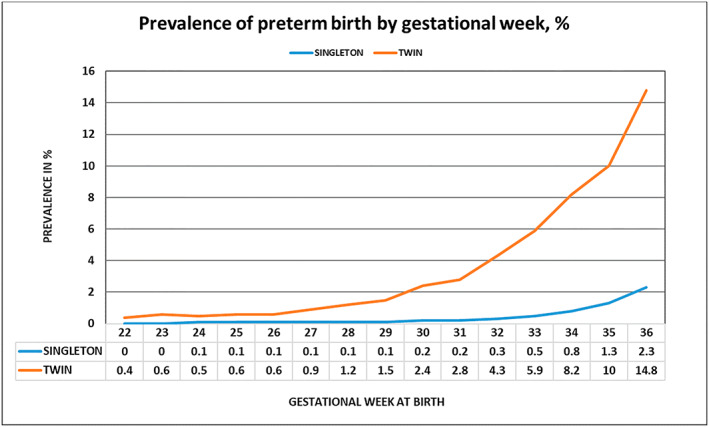
Prevalence (%) of preterm birth by gestational week among nulliparous women with twin and singleton pregnancies (*n* = 33 989, of which 29 211 were singleton pregnancies and 4778 were twin pregnancies).

Of the 481 176 women included, 472 449 (98.2%) had singleton pregnancies and 8727 (1.8%) had twin pregnancies. Preterm birth was increasingly more prevalent among twin pregnancies than among singleton pregnancies for each gestational week up to week 37 (Figure 1). The distribution of risk factors in singleton and twin pregnancies is presented in Table 1.

**TABLE 1:** Prevalence of extremely, very and late preterm birth and factors associated with preterm birth in nulliparous singleton and twin pregnancies (*n* = 481 176)

Factors	Singleton pregnancies, 98.2% (*n* = 472 449)	Twin pregnancies, 1.8% (*n* = 8727)
Gestational age at birth		
Extremely preterm, <28^+0^ weeks	0.4 (1978)	3.6 (312)
Very preterm, 28^+0^–33^+6^ weeks	1.4 (6737)	18.2 (1586)
Late preterm, 34^+0^–36^+6^ weeks	4.3 (20 496)	33.0 (2880)
Term	93.8 (443 238)	45.3 (3949)
Stillbirth	0.4 (1841)	2.3 (197)
Congenital malformations	4.8 (22 685)	13.1 (1146)
Pre‐eclampsia	4.9 (22 919)	16.6 (1447)
Non‐spontaneous onset of birth	24.0 (113 298)	59.7 (5209)
Prelabour rupture of membranes	22.0 (104 117)	22.9 (2000)
Spontaneous onset of birth	76.0 (359 151)	40.3 (3518)
Maternal age		
<25 years	27.3 (129 011)	13.4 (1167)
25–29 years	38.5 (181 917)	31.7 (2767)
30–34 years	25.0 (118 221)	34.6 (3020)
35–39 years	7.8 (36 751)	16.1 (1403)
>39 years	1.4 (6549)	4.2 (370)
Diabetes		
Type 1	0.5 (2196)	0.6 (51)
Type 2	0.3 (1388)	0.6 (55)
Gestational	1.9 (9144)	2.8 (242)
Smoking		
Sometimes	1.7 (7959)	1.4 (124)
Daily	10.4 (48 946)	7.9 (692)
Missing	14.8 (69 929)	15.1 (1316)
In vitro fertilisation	3.6 (17 208)	30.1 (2627)
Maternal country of birth		
Norway	77.5 (366 208)	80.2 (6997)
Europe, EEA	8.6 (40 442)	8.0 (697)
Europe, non‐EEA, Transcaucasia and Central Asia	2.1 (10 093)	2.3 (199)
North America	0.4 (2029)	0.6 (56)
Latin America/Caribbean	1.0 (4688)	0.9 (82)
Middle East/North Africa	2.0 (9414)	1.8 (155)
Sub‐Saharan Africa	2.2 (10 534)	1.5 (132)
South Asia	1.9 (8837)	1.4 (118)
East Asia, Pacific and Oceania	3.2 (15 182)	1.9 (170)
Missing	1.1 (5022)	1.4 (121)
Education		
No/compulsory	14.3 (67 620)	11.0 (961)
Secondary	27.4 (129 281)	27.4 (2391)
Bachelor	39.6 (187 283)	42.0 (3666)
Master/PhD	15.9 (75 304)	17.5 (1528)
Missing	2.7 (12 961)	2.1 (180)
Marital status		
Married/co‐habiting	89.9 (424 929)	92.8 (8095)
Other	10.1 (47 520)	7.2 (632)

### 3.1 Overall prevalence of pregnancy complications

Pregnancy‐related complications associated with preterm birth were differentially distributed in singleton and twin pregnancies in the categories of preterm birth. Stillbirth was significantly more prevalent in twin pregnancies than in singleton pregnancies resulting in very preterm and term births. Congenital malformations were significantly more prevalent in twin pregnancies than in singleton pregnancies for all gestational age categories. Overall, pre‐eclampsia was more than twice as prevalent in twin pregnancies (16.9%) than in singleton pregnancies (4.8%), although this prevalence was greater only for twin pregnancies resulting in late preterm and term births. Overall, non‐spontaneous onset of birth was more than three times more prevalent in twin pregnancies (60.5%) than in singleton pregnancies (18.7%), although this prevalence was significantly greater only for twin pregnancies resulting in late preterm and term births (Table 2).

**TABLE 2:** Prevalence of extremely, very and late preterm birth and term birth in the total population (*n* = 481 176) and after the stepwise exclusion of factors associated with preterm birth

Gestational age category	Extremely preterm, <28 + 0 weeks	Very preterm, 28 + 0–33 + 6 weeks	Late preterm, 34 + 0–36 + 6 weeks	Term, >37 + 0 weeks
Prevalence in % (*n*)				
Total study population, *n* = 481 176	Prevalence in % (*n*)			
Singleton pregnancies, 98.2% (472 449)	0.4% (1978)	1.4% (6737)	4.3% (20 496)	93.8% (443 238)
Twin pregnancies, 1.8% (8727)	3.6% (312)	18.2% (1586)	33.0% (2880)	45.3% (3949)
Stillbirth, 0.4% (2048)	Prevalence in % (95% CI) *n*			
Singleton pregnancies, 0.4% (1851)	30.0% (28.0%–32.1%) 594	4.9% (4.4%–5.4%) 332	1.1% (0.9%–1.2%) 222	0.2% (0.1%–0.2%) 703
Twin pregnancies, 2.3% (197)	25.0% (20.2%–29.8%) 78	3.0% (2.2%–3.9%) 48	1.0% (0.6%–1.3%) 28	1.1% (0.8%–1.4%) 43
Live births, *n* = 479 128	Prevalence in % (*n*)			
Singleton pregnancies, 98.2% (470 598)	0.3% (1384)	1.4% (6405)	4.3% (20 274)	94.0% (442 535)
Twin pregnancies, 1.8% (8530)	2.7% (234)	18.0% (1538)	33.4% (2852)	45.8% (3906)
Congenital malformations 4.9% (23 638)	Prevalence in % (95% CI) *n*			
Singleton pregnancies, 4.8% (22 537)	42.9% (40.3%–45.5%) 594	14.1% (13.3%–15.0%) 906	8.6% (8.2%–9.0%) 1743	4.4% (4.3%–4.4%) 19 294
Twin pregnancies, 12.9% (1101)	62.4% (56.1%–68.6%) 146	19.9% (17.9%–21.9%) 306	10.7% (9.6%–11.9%) 306	8.8% (7.9%–9.7%) 343
Live births without congenital malformations, *n* = 455 490	Prevalence in % (*n*)			
Singleton pregnancies, 98.4% (448 061)	0.2% (790)	1.2% (5499)	4.1% (18 531)	94.5% (423 241)
Twin pregnancies, 1.6% (7429)	1.2% (88)	16.6% (1232)	34.4% (2546)	48.0% (3563)
Pre‐eclampsia, 5.0% (22 588)	Prevalence in % (95% CI) *n*			
Singleton pregnancies, 4.8% (21 335)	22.9% (20.0%–25.8%) 181	30.8% (29.6%–32.0%) 1694	16.2% (15.7%–16.7%) 3001	3.9% (3.8%–3.9%) 16 458
Twin pregnancies, 16.9% (1253)	6.8% (1.4%–12.2%) 6	16.7% (14.6%–18.8%) 206	23.4% (21.7%–25.0%) 595	12.5% (11.4%–13.6%) 446
Live births without congenital malformations or pre‐eclampsia, *n* = 432 902	Prevalence in % (*n*)			
Singleton pregnancies, 98.6% (426 726)	0.1% (609)	0.9% (3804)	3.6% (15 530)	95.3% (406 783)
Twin pregnancies, 1.4% (6176)	1.3% (82)	16.6% (1026)	31.6% (1951)	50.5% (3117)
PROM, 22.4% (97 093)	Prevalence in % (95% CI) *n*			
Singleton pregnancies, 22.4% (95 568)	30.0% (26.4%–33.7%) 183	31.7% (30.2%–33.2%) 1026	36.4% (35.6%–37.1%) 5650	21.8% (21.6%–21.9%) 88 529
Twin pregnancies, 24.7% (1525)	26.8% (17.0%–36.6%) 22	29.7% (26.9%–32.5%) 305	25.7% (23.8%–28.7%) 502	22.3% (20.9%–23.8%) 696
Live births without congenital malformations, pre‐eclampsia or PROM, *n* = 335 809	Prevalence in % (*n*)			
Singleton pregnancies, 98.6% (331 158)	0.1% (426)	0.8% (2598)	3.0% (9880)	96.1% (318 254)
Twin pregnancies, 1.4% (4651)	1.3% (60)	15.5% (721)	31.2% (1449)	52.1% (2421)
Non‐spontaneous onset, 19.3% (64 654)	Prevalence in % (95% CI) *n*			
Singleton pregnancies, 18.7% (61 839)	17.4% (13.8%–21.0%) 74	31.6% (29.8%–33.4%) 820	22.5% (21.7%–23.4%) 2227	18.5% (18.3%–18.6%) 58 718
Twin pregnancies, 60.5% (2815)	20.0% (9.58%–30.4%) 12	34.8% (31.3%–38.3%) 251	48.4% (45.9%–51.0%) 702	76.4% (74.7%–78.1%) 1850
Live births with spontaneous labour onset, without congenital malformations, pre‐eclampsia or PROM, *n* = 271 155	Prevalence in % (95% CI) *n*			
Singleton pregnancies, 99.3% (269 319)	0.1% (0.1%–0.1%) 352	0.7% (0.6%–0.7%) 1778	2.8% (2.8%–2.9%) 7653	96.4% (96.3%–96.4%) 259 536
Twin pregnancies, 0.7% (1836)	2.6% (1.9%–3.3%) 48	25.6% (23.6%–27.6%) 470	40.7% (38.4%–42.9%) 747	31.1% (29.0%–33.2%) 571

### 3.2 Extremely preterm birth (<28^+0^ weeks of gestation)

In the entire study population, the prevalence of extremely preterm birth was 0.4% in singleton pregnancies and 3.6% in twin pregnancies (Table 1). Among women experiencing extremely preterm live birth with no fetal congenital malformation, pre‐eclampsia was significantly more prevalent in singleton pregnancies (22.9%, 95% CI 20.0%–25.8%) than in twin pregnancies (6.8%, 95% CI 1.4%–12.2%) (Table 2). Among women experiencing extremely preterm live birth with no fetal congenital malformation or pre‐eclampsia, the prevalence of PROM did not differ significantly between singleton pregnancies (30.0%, 95% CI 26.4%–33.7%) and twin pregnancies (26.8%, 95% CI 17.0%–36.6%) (Table 2). Among women experiencing live spontaneous onset of birth with no fetal congenital malformation, pre‐eclampsia or PROM, extremely preterm birth was significantly more prevalent in twin pregnancies (2.6%, 95% CI 1.9%–3.3%) than in singleton pregnancies (0.1%, 95% CI 0.1%–0.1%) (Table 2).

In the entire study population (model 1), the odds of extremely preterm birth were about eight times greater for twin pregnancies than for singleton pregnancies (aOR 7.79, 95% CI 6.83–8.89) (Table 3). Among women with spontaneous live births with no malformation, pre‐eclampsia or PROM (model 2), the odds of extremely preterm birth were about 19 times greater for twin pregnancies than for singleton pregnancies (aOR 19.33, 95% CI 13.93–26.84).

**TABLE 3:** Risk of extremely, very and late preterm birth in nulliparous women with twin pregnancies compared with nulliparous women with singleton pregnancies

	Model 1, all cases		Model 2, live birth, spontaneous^a^	
	Crude analysis, OR (95% CI)	Multivariable analysis, aOR (95% CI)^b^	Crude analysis, OR (95% CI)	Multivariable analysis, aOR (95% CI)^b^
Extremely preterm birth, <28 weeks of gestation				
Singleton pregnancies	Ref.	Ref.	Ref.	Ref.
Twin pregnancies	8.82 (7.81–9.96)	7.79 (6.83–8.89)	20.51 (15.12–27.83)	19.33 (13.93–26.84)
Very preterm birth, 28–33^+6^ weeks of gestation				
Singleton pregnancies	Ref.	Ref.	Ref.	Ref.
Twin pregnancies	15.99 (15.06–16.97)	15.05 (14.11–16.06)	53.59 (47.76–60.13)	52.54 (46.34–59.56)
Late preterm birth, 34–36^+6^ weeks of gestation				
Singleton pregnancies	Ref.	Ref.	Ref.	Ref.
Twin pregnancies	15.77 (15.00–16.59)	15.31 (14.52–16.14)	44.36 (39.69–49.59)	43.47 (38.71–48.82)

^a^ Spontaneous onset of birth, live births with no congenital malformation, pre‐eclampsia or prelabour rupture of membranes.

^b^ Adjusted for maternal age, marital status, education, maternal country of birth, diabetes, smoking and in vitro fertilization.

### 3.3 Very preterm birth (28^+0^–33^+6^ weeks of gestation)

In the entire study population, the prevalence of very preterm birth was 1.4% in singleton pregnancies and 18.2% in twin pregnancies (Table 1). Among women experiencing very preterm live birth with no fetal congenital malformation, pre‐eclampsia was significantly more prevalent in singleton pregnancies (30.8%, 95% CI 29.6%–32.0%) than in twin pregnancies (16.7%, 95% CI 14.6%–18.8%) (Table 2). Among women experiencing very preterm live birth with no fetal congenital malformation or pre‐eclampsia, the prevalence of PROM did not differ significantly between singleton pregnancies (31.7%, 95% CI 30.2%–33.2%) and twin pregnancies (29.7%, 95% CI 26.9%–32.5%) (Table 2). Among women experiencing live spontaneous birth with no fetal congenital malformation, pre‐eclampsia or PROM, very preterm birth was significantly more prevalent in twin pregnancies (25.6%, 95% CI 23.6%–27.6%) than in singleton pregnancies (0.7%, 95% CI 0.6%–0.7%) (Table 2).

In the entire study population, the odds of very preterm birth were 15 times greater for twin pregnancies than for singleton pregnancies (aOR 15.0, 95% CI 14.11–16.06) (Table 3). Among those with spontaneous live births with no fetal congenital malformation, pre‐eclampsia or PROM, the odds of preterm birth were about 53 times greater for twin pregnancies than for singleton pregnancies (aOR 52.54, 95% CI 46.34–59.56).

### 3.4 Late preterm birth (34^+0^–36^+6^ weeks of gestation)

In the entire study population, the prevalence of late preterm birth was 4.3% in singleton pregnancies and 33.0% in twin pregnancies (Table 1). Among women experiencing late preterm live birth with no fetal congenital malformation, pre‐eclampsia was significantly more prevalent in twin pregnancies (23.4%, 95% CI 21.7%–25.0%) than in singleton pregnancies (16.2%, 95% CI 15.7%–16.7%) (Table 2). Among women experiencing late preterm live birth with no fetal congenital malformation or pre‐eclampsia, PROM was significantly more prevalent in singleton pregnancies (36.4%, 95% CI 35.6%–37.1%) than in twin pregnancies (25.7%, 95% CI 23.8%–28.7%). Among women experiencing spontaneous live birth with no fetal congenital malformation, pre‐eclampsia or PROM, late preterm birth was significantly more prevalent in twin pregnancies (40.7%, 95% CI 38.4%–42.9%) than in singleton pregnancies (2.8%, 95% CI 2.8%–2.9%).

In the entire study population, the odds of late preterm birth were about 15 times greater for twin pregnancies than for singleton pregnancies (aOR 15.31, 95% CI 14.52–16.14) (Table 3). Among women with spontaneous live births with no fetal congenital malformation, pre‐eclampsia or PROM, the odds of late preterm birth were about 43 times greater for twin pregnancies than for singleton pregnancies (aOR 43.47, 95% CI 38.71–48.82).

## 4 DISCUSSION

### 4.1 Main findings

Preterm birth was more frequent in twin pregnancies (54.7%) than in singleton pregnancies (6.1%) of nulliparous women. Prevalence of pregnancy‐related complications differed between singleton and twin pregnancies. Overall, pre‐eclampsia was between three and four times more prevalent in twin pregnancies, but the role of pre‐eclampsia as a contributor to preterm birth seemed to differ in singleton and twin pregnancies according to gestational age. Pre‐eclampsia was more prevalent in singleton pregnancies resulting in extremely and very preterm births, but was more prevalent in twin pregnancies resulting in births from 34 weeks of gestation onwards. PROM was distributed equally among twin and singleton pregnancies leading to preterm birth. When obstetric complications associated with preterm birth were excluded from the analysis, the ORs for extremely, very and late preterm birth in twin pregnancies compared with singleton pregnancies increased dramatically from the range of 8–15 to the range of 19–53.

### 4.2 Strengths and limitations

This study was based on data from MBRN, a mandatory register containing data on all births occurring in Norway. As antenatal care in Norway is standardised, with predefined variables reported with high coverage of notification, the risk of bias was limited. The MBRN is known to be a reliable source of data suitable for research.^11^ A validation study found the information on gestational age and birthweight to be very good.^12^ As a large number of births were available for analyses, reliable prevalence and risk estimates for less frequent outcomes, such as extremely preterm birth, could be calculated. This enabled us to show differences in pregnancy‐related complications occurring in three categories of preterm birth in twin and singleton pregnancies.

As predefined variables are recorded in the MBRN, data on some possibly relevant factors, such as infections and cervical length, were not available. Chorionicity is known to be associated with preterm birth in twin pregnancies.^13^ Twin pregnancy chorionicity is not registered in the MBRN. The main objective in this study was, however, to compare singleton pregnancies with all twin pregnancies. In addition, registry data may be affected by misclassification. However, because of the notification format (checkboxes), under‐reporting leading to weaker associations is more likely than over‐reporting.

### 4.3 Interpretation

In this study, pre‐eclampsia in extremely and very preterm births was less prevalent in twin pregnancies than in singleton pregnancies, which is a novel finding. Twin pregnancy is a known risk factor for hypertensive disorders of pregnancy.^4^ The increased prevalence and three‐ to four‐fold greater odds of pre‐eclampsia in twin pregnancies compared with singleton pregnancies is well established. In a population‐based study conducted with MBRN data, Laine et al. observed four‐fold greater odds of hypertensive disorders in twin pregnancies compared with singleton pregnancies.^14^ In line with our findings, Francisco et al. reported increasing risk of preterm pre‐eclampsia with increasing gestational age in twin pregnancies relative to singleton pregnancies,^15^ and Aviram et al. reported that hypertensive disorders of pregnancy were a more prominent cause of preterm birth in singleton pregnancies than in twin pregnancies.^16^ When reviewing research on twin pregnancy and hypertensive disorders, Wang et al. concluded that the pregnancy process, including the pathophysiology of pre‐eclampsia, differs between twin and singleton gestations.^17^ These studies indicate that the aetiology of preterm birth differs between twin and singleton pregnancies, with lower rates of preterm hypertensive disorder in twin pregnancies being a consequence of lower gestational age at delivery in twin pregnancies.

Kalafat et al. showed that 150 mg/day of aspirin is more efficient than 75 mg/day of aspirin in preventing hypertensive disorders in twin pregnancies.^18^ Ye et al. found that 100 mg/day of acetylsalicylic acid (ASA) significantly decreased the risks of pre‐eclampsia and preterm birth before 34 weeks of gestation in twin pregnancies.^19^ These findings indicate that twin pregnancy is a single high‐risk factor for pre‐eclampsia, not just a moderate risk factor, as is currently stated in the UK National Institute for Health and Care Excellence (NICE) guidelines.^20^ The high prevalence of pre‐eclampsia among twin pregnancies in our study, regardless of gestational age at birth, also supports the strategy of offering prophylactic ASA to all women with twin pregnancies, even without other risk factors.

We found that the rates of PROM were similar in singleton and twin pregnancies, except that PROM occurred significantly more frequently in singleton pregnancies resulting in late preterm birth. Few studies have involved an examination of the prevalence of PROM in singleton and twin gestations, and the results reported are conflicting. Barinov et al. found that PROM was more prevalent in singleton than in twin pregnancies resulting in preterm birth.^21^ Pakrashi et al. found that PROM increased with increasing gestation number and decreasing gestational age at birth in a population‐based study conducted in the USA.^22^ Ruijwetpongstorn et al. reported no significant difference in the PROM rate between twin and singleton gestations.^23^ Further large studies are needed to establish the role of PROM in preterm twin and singleton births.

## 5 CONCLUSION

Singleton and twin pregnancies seem to have different pathways leading to preterm birth. Stillbirth, congenital malformations and pre‐eclampsia were more prevalent in twin pregnancies than in singleton pregnancies in the entire population. However, pre‐eclampsia was more prevalent in singleton pregnancies than in twin pregnancies ending in extremely or very preterm births. These findings indicate that hypertensive disorders play different roles in twin and singleton preterm births, probably because preterm twin births occur at an earlier gestation for other reasons. The role of twin pregnancy as a risk factor for preterm birth increased dramatically when removing other major risk factors for preterm birth. Tailored antenatal care should be offered to women expecting twins, including considering twin pregnancy a sufficient risk factor to offer prophylactic ASA. In addition, this study corroborates that single‐embryo transfer is the appropriate strategy in artificial reproductive therapy, in line with the latest recommendations from the International Federation of Gynaecology and Obstetrics.^24^

## AUTHOR CONTRIBUTIONS

TT, SR, ÅV, LS, GM, MS and KL contributed to the planning and design of the study, interpretation of the data and critical revision of the article. TT drafted the article and performed the analyses.

## FUNDING INFORMATION

The Norwegian SIDS and Stillbirth Society provided financial support for the retrieval of data files from the registries (grant no. 554.04/14).

## CONFLICT OF INTERESTS

None declared. Completed disclosure of interests form available to view online as supporting information.

## ETHICS APPROVAL

This study is part of the PURPLE Study, approved by the Regional Committee for Medical Research Ethics in South East Norway in 2015 (2015/681), and was evaluated by the Institutional Personal Data Officer of Oslo University Hospital. It was conducted in accordance with Norwegian health research legislation. All data were anonymised by registry management staff. Patient consent is not required for research using data from the MBRN.

## Supporting information


Appendix S1.

Appendix S2.

Appendix S3.

Appendix S4.

Appendix S5.

Appendix S6.

Appendix S7.

## Data Availability

The data that support the findings of this study are available from the Medical Birth Registry of Norway and Statistics Norway. Restrictions apply to the availability of these data, which were used under license for this study.
